# An Orally Administered Nonpathogenic Attenuated Vaccine Virus Can Be Used to Control SARS-CoV-2 Infection: A Complementary Plan B to COVID-19 Vaccination

**DOI:** 10.7759/cureus.28467

**Published:** 2022-08-27

**Authors:** Tibor Bakacs, Volker Sandig, Imre Kovesdi

**Affiliations:** 1 Department of Probability, Alfréd Rényi Institute of Mathematics; The Eötvös Loránd Research Network, Budapest, HUN; 2 Chief Scientific Officer, ProBioGen AG, Berlin, DEU; 3 Chief Scientific Officer, Unleash Immuno Oncolytics Inc., St. Louis, USA

**Keywords:** paul ehrlich institute, nih-activ, oral treatment, ibdv, infectious bursal disease virus, superinfection therapy, interferon beta, sars-cov-2, severe acute respiratory syndrome coronavirus-2, covid-19 retro

## Abstract

Background

Coronavirus disease 2019 (COVID-19) vaccination has substantially altered the course of the pandemic, saving tens of millions of lives globally. The problem is that despite such spectacular results, vaccination alone will not be able to control the COVID-19 pandemic because of the rapid evolution of the severe acute respiratory syndrome coronavirus 2 (SARS-CoV-2) even in vaccinated human populations. Therefore, the development of a post-infection, broad-based, orally administered antiviral therapy that would complement vaccination efforts is urgently needed.

Methodology

The so-called viral superinfection therapy (SIT) administers a nonpathogenic attenuated double-stranded RNA (dsRNA) vaccine virus drug candidate, the infectious bursal disease virus serotype R903/78 (IBDV-R903/78) that activates the interferon (IFN) genes, which are the natural, antiviral defense system of host cells.

Results

Here we present two cases of properly vaccinated (with BNT162b2-Pfizer) and booster-dosed COVID-19 patients with vaccine breakthrough infections whose disease duration was shortened to a few days by oral SIT.

Conclusions

SIT has already been demonstrated to be safe and effective against five different families of viruses, hepatitis A virus, hepatitis B virus, hepatitis C virus, SARS-CoV-2, and herpes zoster virus. The R903/78 drug candidate is simple to manufacture and easy to administer in an outpatient setting. The expected cost of SIT will be affordable even in resource-limited countries.

## Introduction

Vaccination has had a substantial impact on the trajectory of the coronavirus disease 2019 (COVID-19) pandemic. A global reduction of 63% in total deaths (19.8 million from 31.4 million) during the first year of COVID-19 vaccination was achieved [1]. In the largest vaccination program in history, by December 8, 2021, 55.9% of the global population was estimated to have received at least one dose, 45.5% two doses, and 4.3% have received a booster dose of a COVID-19 vaccine. The problem is that despite such huge efforts and spectacular results, vaccination alone will not be able to control the COVID-19 pandemic.

Genome sequencing showed that severe acute respiratory syndrome coronavirus 2 (SARS-CoV-2) is picking up about two single-letter mutations per month. The assumption that viruses evolve to become milder is a myth. The high circulation of the Delta and Omicron variants aided by inequitable vaccine rollouts and minimal control measures in some wealthy countries offer fertile ground for SARS-CoV-2 to take additional surprising evolutionary leaps. Indeed, there is a possibility that SARS-CoV-2 could become more severe or evade current vaccines by recombining with other coronaviruses. Circulation in animal reservoirs could also bring unexpected changes [2].

In fact, scientists have no idea what comes next [3]. A new variant different from Omicron may already be developing, perhaps in a chronically infected patient. It seems that Louis Pasteur’s famous warning, “…it is the microbes who will have the last word,” a century and a half later is still correct.

According to the World Health Organization (WHO), COVID-19 vaccine hesitancy is one of the top threats that inhibits global control of SARS-CoV-2 infections. Almost one‑third of people had no intention of receiving any COVID‑19 vaccine [4]. Considering the polarized attitude toward vaccination, it will be very difficult to achieve herd immunity to block transmission and reduce the socioeconomic burden of the disease.

Clearly, new drugs against SARS-CoV-2 will be needed to counter the looming threat of resistance [5]. Developing broad-spectrum drugs will take significant public and private investment. Consistent with this, the United States provided USD 1.2 billion for basic research on developing antivirals for seven virus families [5].

Fortunately, a broad-spectrum, orally administered, and affordable post-infection antiviral drug candidate has already been demonstrated to be safe and effective against five different families of viruses, including SARS-CoV-2. The drug candidate is an attenuated, live avian vaccine virus, the infectious bursal disease virus (IBDV), which is harmless to people with healthy immune systems (while the safety of IBDV should yet be established in immunocompromised patients). The objective of this paper, therefore, is to stimulate a meaningful discussion as to how this innovative post-infection therapy can complement COVID-19 vaccination.

Rationale and proof-of-concept of the viral superinfection therapy (SIT)

Currently, antiviral drugs are developed against a single virus. However, more than 200 viruses are known to infect humans, while treatments are available only against a few of them. Therefore, the “one drug, one bug” approach should be complemented with a “one drug, multiple bugs” strategy. This objective could be achieved if the host rather than the virus is targeted. The orally administered IBDV, for example, activates interferon (IFN) genes, which are the natural, antiviral defense system of host cells following exposure to viral infection [6].

Through a family of innate pattern-recognition receptors, such as the Toll-like receptors (TLRs), cells recognize pathogen-associated molecular patterns (PAMPs) [7]. The double-stranded RNA (dsRNA) is a molecular pattern, which is associated with viral infections. Most viruses produce dsRNA at some point during their replication cycle activating the IFN gene defense system. The IBDV genome is composed of dsRNA, which induces one of the strongest IFN responses among viruses [8].

Therefore, superinfection of patients by the nonpathogenic attenuated IBDV interferes with the replication of disease-causing viruses. IBDV has been demonstrated to be effective against hepatitis A virus (HAV) infection in marmoset monkeys. More importantly, IBDV has been proven to be safe and effective in 50 patients with four different viral infections (hepatitis B virus (HBV), hepatitis C virus (HCV), SARS‑CoV‑2, and herpes zoster virus (HZV)) [6]. IBDV’s IFN activation properties make it a promising drug candidate to counter SARS-CoV-2 infection, which is extremely sensitive to IFN [9].

The safety aspects of the IBDV drug candidate

For any virus to jump between donor and recipient host species and establish a productive infection depends on the relatedness of these species. Key components of the virus-host interaction in birds and mammals diverged along with their hosts more than 200 million years ago such that 13 mutations may be required for avian influenza viruses to establish productive infections in humans [10]. To pre-adapt to humans, the influenza virus requires an intermediate host (the swine) for such a gigantic jump. IBDV, however, does not have such a natural host. Consistent with this, no zoonosis cases were ever reported over the past 50 years during IBDV mass vaccination programs in poultry. Notwithstanding, even a very low risk of zoonosis is a legitimate regulatory concern. Therefore, reverse genetics technology was used to create the IBDV-R903/78 drug candidate, providing an easily testable homogenous starting material to ensure batch-to-batch consistency without the need for repeated plaque purification [11].

No serious side effects were observed during IBDV superinfection therapy even in parenchymal decompensated moribund hepatitis patients [12-14]. This is in stark contrast to systemic IFN-based therapy, which is associated with a wide array of adverse effects that may require dose modification or even discontinuation of therapy. One possible explanation could be that receptors for type I and II IFNs are found on the surface of most cell types such that systemic IFN therapy has an almost ubiquitous nature of signaling. While following the interaction of IBDV with appropriate cells, its dsRNA is recognized by specific receptors (e.g., TLR3, TLR10), which activate several gene families from within [6]. TLR10 is a novel dsRNA nucleotide-sensing receptor with anti-inflammatory properties. Lee et al. demonstrated crosstalk between TLR10 and TLR3, which opens up a new concept in the regulation of IFN response [15]. In this context, it is important to emphasize that IBDV therapy never induced an excessive release of pro-inflammatory cytokines, even in decompensated hepatitis patients with high-level viremia, which is one of the key drivers of cytokine storms [8]. It is tempting to speculate that the anti-inflammatory properties of TLR10 contribute to the safety of IBDV therapy.

## Materials and methods

The manufacturing of the IBDV-R903/78 drug candidate

For manufacturing the R903/78 drug candidate, the continuous AGE1.CR.PIX cell line has been chosen. AGE1.CR.PIX was created by immortalization of the Muscovy embryonic duck retina cells with the E1A and E1B genes. A combination of E1 genes (E1A, E1B 55k, and E1B 19K) from human adenovirus type 5 was chosen for pharmaceutically safe immortalization. This method is consistent with the “defined-risk approach” because the immortalizing genes are known and traceable [16]. The cell line has been banked under Good Medical Practice (GMP) conditions. Specifically, isolation and immortalization of primary cells from the Muscovy duck embryo retina were performed in a dedicated GMP unit under sterile conditions with defined solutions and media. The AGE1.CR.PIX cell line has been shown to be free of adventitious agents and used for the production of clinical-grade, live-attenuated, vectored vaccines based on Modified Vaccinia virus Ankara (MVA). AGE1.CR.PIX has been selected for its high permissivity and titers exceeding other cell lines by 100-1,000-fold reaching levels between 10^9^ and 10^10^ infectious units (IU)/mL. Assuming a typical clinical dose of 10^7^ IU, 100,000 to 1,000,000 doses can be produced in 1 L fermentation volume. For the manufacture of parenteral live vaccines, a suitable purification strategy is applied to reduce host cell protein contamination and to assure levels of host cell DNA below 10 ng/dose. Oral or intranasal application, which is planned for R903/78, requires less stringent reductions of host cell DNA and protein. The manufacturing process is furthermore simplified as R903/78 is secreted into the cell culture supernatants not requiring lysing of the cells greatly reducing downstream purification complexity and expense. The expected high virus concentration in the yield combined with oral delivery allows for a simple formulation methodology.

Phase I safety clinical protocol of the R903/78 drug candidate of SIT for the treatment of COVID-19 patients with mild symptoms

The primary objective will be to determine the safety of the R903/78 oral biologic drug candidate. The secondary objective is to determine the effect of the biologic agent on SARS-CoV-2 RNA, immunologic parameters, and the clinical condition of the patients. A small Phase I safety/dose-finding clinical trial involving 18 COVID-19 patients with mild symptoms is planned. All patients will receive the R903/78 agent orally once a day for seven days: 1.0 × 10^6^ IU in the low-dose group, 1.0 × 10^7^ IU in the intermediate-dose group, and 1.0 × 10^8^ IU in the high-dose group [6]. The starting dose is supported by good laboratory practice (GLP) toxicity studies. Our experience with five COVID-19 patients and one HZV patient indicates that one-week IBDV treatment is sufficient in these acute indications.

The criteria for dosage escalation will be based on the absence of dose-limiting toxicity in four subjects within each group on the R903/78 agent. Safety data will be reviewed on an ongoing basis. Dose escalation from one dose level to the next may be allowed one week after the fourth patient in the lower-dose group has been treated and if no dose-limiting side effects have been observed. Primary endpoints will be safety and tolerability with secondary endpoints such as viral RNA levels and improvement of clinical symptoms.

## Results

SIT of breakthrough COVID-19 infection in two vaccinated booster-dosed 75 and 73-year-old medical doctors with mild symptoms

Here, we present cases of vaccine breakthrough infections in two medical doctors (T.B. [75 years], who is the first author of this paper, and his wife E.M. [73 years]; consent to the treatment and publication of patient information were granted by both patients). They were properly vaccinated with two doses of Pfizer-BioNTech COVID-19 vaccines (BNT162b2-Pfizer). Then, T.B. received two booster doses, while E.M. received one booster dose of the Pfizer-BioNTech COVID-19 vaccine. None of the patients experienced typical or severe COVID-19 symptoms, except mild fever and fatigue.

First, T.B. became ill with mild fever and fatigue. Because the fatigue continued for several days, he performed the ViVaDiag SARS-CoV-2 Ag Rapid Test on March 11, 2022, which proved to be positive. Therefore, T.B. started oral SIT with an attenuated IBDV veterinary vaccine virus, which is commercially available in Hungary. One lyophilized ampoule (1 × 10^6^ IU = 10^6^ TCID_50_ [TCID = tissue culture infective dose]) was dissolved in tap water as instructed by the manufacturer (Ceva-Phylaxia Zrt) and then swallowed in the morning and the same dose again in the evening on March 12, 13, 14, 2022 (6 × 10^6^ IU in total). The sub-febrile state and fatigue vanished by the second day of treatment. The patient experienced no side effects. The repeated ViVaDiag SARS-CoV-2 Ag Rapid Test on March 15, 2022, proved to be negative. The patient felt physically completely recovered. He performed a functional cardiovascular test, which was a slow pace of 30 minutes of running with an average pulse rate of 130/minute. This is his usual running pace. A couple of hours later his resting pulse rate returned to 58/minute with an SpO_2_ of 96%. Importantly, four weeks after the IBDV-SIT, the patient’s serum tested positive for anti-IBDV neutralizing antibodies [17]. This finding confirmed results obtained in animal models following oral administration of IBDV. Please note that humans are not a natural host of IBDV. Therefore, the appearance of neutralizing antibodies after oral administration of the live-attenuated IBDV demonstrates its interaction with the patient’s immune system. In this context, it is important to note that multiple oral IBDV administrations generated high levels of neutralizing antibodies in mice. Breakthrough infection was, however, achieved by maintaining oral IBDV administration [17]. This is consistent with clinical observations in decompensated hepatitis patients when large doses of the viral preparation were administered continuously over a long period for the maintenance of “artificial viremia” [11,13].

E.M. was shivering, had a mild fever and cough, and complained about fatigue, myalgias, nausea, vomiting, headache, weakness, and rhinorrhea on March 15, 2022 (T.B. and E.M. are living together). Her ViVaDiag SARS-CoV-2 Ag Rapid Test proved positive on March 15, 2022. Importantly, during the previous days of her husband’s disease, her ViVaDiag SARS-CoV-2 Ag Rapid Tests proved to be repeatedly negative, indicating that most probably she contracted COVID-19 from T.B. Therefore, she started oral SIT with the same attenuated IBDV veterinary vaccine virus that her husband used before, administering 1 × 10^6^ IU dose first at noon, and then the same 1 × 10^6^ IU dose in the evening. The mild fever, cough, running nose, and fatigue vanished by the second day. The patient had another 1 × 10^6^ IU dose of IBDV the next morning, on March 16, 2022, but she refused further treatment because she felt cured (after a 3 × 10^6^ IU dose in total). The patient experienced no side effects. The repeated ViVaDiag SARS-CoV-2 Ag Rapid Test on March 16, 2022, was still positive, but a day later on March 17, 2022, the Rapid Ag Test became negative. The patient felt physically completely recovered. Therefore, she performed a functional cardiovascular test on March 17, 2022, which was a slow pace of 3.4 km running during which her average pulse rate was 113/minute. A few hours later her resting pulse rate returned to 76/minute with an SpO_2_ of 96%.

## Discussion

Originally, viral interference referred to the phenomenon when an established viral infection suppressed the replication of a coinfecting virus. While in HBV and HCV-infected patients, it was observed that the replication of the coinfecting virus could also be dominant over the replication of the infecting virus. Dominance can alternate between the two hepatitis viruses but, usually, HBV appears to be suppressed by HCV. Nevertheless, when HBV infection is suppressed by HCV infection, the disease persists and hepatitis remains [18].

Natural viral dominance can be interrupted by direct-acting antiviral (DAA) HCV therapy in patients with HBV/HCV coinfection. Following DAA therapy, HBV reactivation, an abrupt increase in HBV replication, can occur that may result in clinically significant hepatitis. HBV reactivation is a newly identified safety concern in HBV/HCV-coinfected patients who are treated with DAAs. The Food and Drug Administration (FDA), therefore, issued a black box warning that all patients who are being treated with DAA agents for HCV infection must undergo HBV panel testing [19].

When the coinfecting virus does not cause disease, the clinical course is more favorable. Hepatitis G virus (HGV) and GB Virus type C (GBV-C) were identified in the mid-1990s and were proved to be identical. Because this virus does not cause hepatitis despite sharing genome organization with HCV, the virus was renamed “human pegivirus 1” or HPgV-1 for “persistent G” virus. Several studies found that HPgV-1 coinfection was associated with prolonged survival in people living with HIV [20].

**Figure 1:**
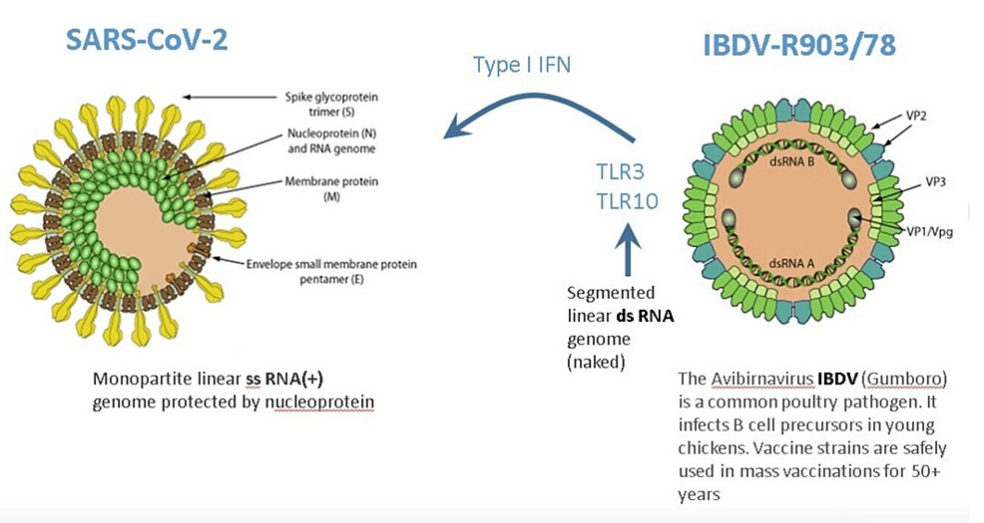
Superinfection with a harmless virus will induce antiviral host defenses. Innate immunity represents the only protection against a virus in a naïve population. Image credits: Tibor Bakacs, Volker Sandig, Imre Kovesdi. SARS-CoV-2: severe acute respiratory syndrome coronavirus 2; IFN: interferon; IBDV: infectious bursal disease virus

Natural coinfection can be imitated by intentionally coinfecting patients with a nonpathogenic virus. Intentional coinfection is called SIT. SIT was developed in the 90s using a conventionally produced attenuated avian vaccine virus, the IBDV, which is a potent activator of the IFN-dependent antiviral gene program (Figure 1).

IBDV-SIT has been demonstrated to be safe and effective in 42 acute HBV and HCV patients but also in four decompensated moribund HBV and HCV patients [12-14]. Further, IBDV-SIT was safe and effective in three COVID-19 patients [6] and in an HZV patient [21] (Table 1).

**Table 1:** IBDV has been proven clinically safe and effective against five different viruses in acute and chronic diseases COVID-19: coronavirus disease 2019; SARS-CoV-2: severe acute respiratory syndrome coronavirus 2; IBDV: infectious bursal disease virus

Type of virus	Number of patients	References
Hepatitis A virus	Marmoset monkeys	[11]
Hepatitis B virus	20 acute and 2 decompensated patients	[12,13]
Hepatitis C virus	22 acute and 2 decompensated patients	[12,13]
COVID-19 disease (SARS-CoV-2)	5 acute patients	[6] and the current article
Shingles (herpes zoster virus)	1 acute patient	[21]

Clearly, without controls and with N = 2 it is hard to tell whether the recovery of COVID-19 patients can be attributed to IBDV. However, before COVID-19 vaccines became available, three other COVID-19 patients had also recovered within a few days, including a patient with severe shortness of breath using similar dosages of IBDV (see also below) [6].

In the above context, it is important to recall that stimulation of innate immunity by live-attenuated oral enteroviral IFN-inducing vaccine strains (LEV) in large-scale clinical studies involving about 320,000 people provided temporary protection against seasonal influenza and acute respiratory infections (ARI) between 1968 and 1971 in 16 regions of three republics of the former Soviet Union. LEV reduced the morbidity of influenza and ARI by 3.2-fold on average [22]. Recently, Chumakov et al. proposed that live-attenuated vaccines, in general, and oral poliovirus vaccine (OPV), in particular, could provide temporary protection against COVID-19 by the IFN-mediated stimulation of innate immunity. The authors stated that this strategy may even have an advantage over specific vaccines if SARS-CoV-2 undergoes mutations that could lead to a loss of vaccine efficacy [23]. Recently the proof-of-concept was provided by Yagovkina et al. demonstrating that immunization with bivalent OPV reduced the number of laboratory-confirmed COVID-19 cases, consistent with the original hypothesis that live-attenuated vaccine viruses induce nonspecific protection against off-target infections [24].

By modeling millions of viral sequences across thousands of regions, Obermeyer et al. demonstrated that the SARS-CoV-2 pandemic repeated waves were driven by the emergence of new lineages with higher fitness, affecting their basic reproduction number (R_0_), and their ability to evade existing immunity [25]. More than two years of pandemic experience has demonstrated that circulating lineages are regularly displaced by the sudden emergence of markedly fitter variants. Such rapid viral evolution cannot be controlled by vaccination alone.

Viruses have the unique ability to affect hundreds of genes with minimal genomic burden [26]. The small dsRNA genome of IBDV, for example, activates many IFN-related genes (toll-like receptor 3 (Tlr3), toll-like receptor 9 (Tlr9), Z-DNA binding protein 1 (Zbp1), IFN-activated gene 204 (Ifi204), IFN-gamma (IFN-g)) [8]. In the absence of adaptive immunity, the IFN system can control most, if not all, virus infections. Therefore, IBDV superinfection may provide a strategy to control viral infections, particularly COVID-19 disease. We are not alone with such a proposal. In a recent Frontiers in Immunology’s Research Topic entitled, “Fighting fire with fire: using nonpathogenic viruses to control unrelated infections,” several publications suggested similar strategies exploiting nonpathogenic viruses for antiviral therapy (e.g., oral polio vaccine virus [24], human pegivirus type 1 (HPgV-1) [20]).

While scientists hunt for drugs to treat mild COVID-19 disease, both because of broad public health benefits and because long quarantine periods disrupt many people’s lives [27], oral IBDV superinfection therapy has safely and successfully treated unvaccinated [6] and vaccinated COVID-19 patients with breakthrough infections shortening disease duration to a few days. The fast clearance of the SARS-CoV-2 virus after SIT (rapid Ag test became negative within four and two days, respectively) argues for the efficacy of IBDV treatment because untreated vaccine breakthrough infections exhibit, on average, 6.2 days of virus clearance time [28].

Importantly, an expert team of the US National Institutes of Health-sponsored ACTIV public-private partnership, arguably one of the most important organizations fighting the COVID-19 pandemic, came to the conclusion that the IBDV-R903/78 drug candidate shows merit as a potential treatment for COVID-19. In preparation for the Phase I clinical study for early COVID-19 patients, IBDV superinfection was discussed with the German Paul Ehrlich Institute. Therefore, if it is approved, the IBDV-based SIT could be an important drug for the treatment of mild-to-moderate COVID-19 disease.

We need a complementary therapeutic plan “B” to vaccination as soon as possible to combat mild-to-moderate SARS-CoV-2 infections. Therefore, a discussion about the further development of the broad-based orally administered post-infection IBDV superinfection antiviral therapy would be useful.

A major limitation of this study is that the proof-of-principle of SIT in COVID-19 disease was thus far demonstrated by case studies only. While case reports lack statistical analyses, they can provide clinical insights that may be missed in clinical trials [29]. For example, the first advanced leukemia patient who was cured by the experimental chimeric antigen receptor (CAR) T cell therapy [30] paved the way for revolutionary medical advances. In this context, it is important to note that even before COVID-19 vaccines became available, SIT proved to be safe and effective in three unvaccinated patients with typical COVID-19 symptoms, including severe cough, fever, fatigue, muscle aches, headache, loss of smell, and shortness of breath. All three patients recovered within a few days [6]. To address this limitation, we are preparing a Phase I clinical study of IBDV superinfection for early COVID-19 patients, which has already been discussed with the German Paul Ehrlich Institute. Because one of our COVID-19 patients (unpublished case report) with progressively severe cough and shortness of breath, with deteriorating general condition (the patient was terrified of dying), became totally asymptomatic within seven days of IBDV treatment (7 × 10^6^ IU in total), it is tempting to speculate that the IBDV R903/78 drug candidate with its IFN activation properties would be promising as anti-SARS-CoV-2 therapy even in severe COVID-19 patients.

## Conclusions

Due to the rapid evolution SARS-CoV-2 virus in human populations including vaccinated people, there is an urgent need for a complementary therapeutic plan “B” to control the COVID-19 pandemic. Here, we present an innovative post-infection strategy: antiviral SIT. SIT orally administers a nonpathogenic attenuated live avian vaccine virus, IBDV, which induces IFNs by its dsRNA genome from within the host cells. The proof of principle of IBDV-SIT has been demonstrated in five different viral infections, HAV, HBV, HCV, SARS-CoV-2, and HZV. The R903/78 drug candidate is simple to manufacture and easy to administer in an outpatient setting. The expected cost of SIT will be affordable even in resource-limited countries.
